# Alveolar rhabdomyosarcoma of the paranasal sinuses with delayed diagnosis in a resource-constrained clinical setting: a case report

**DOI:** 10.1093/omcr/omaf256

**Published:** 2025-12-26

**Authors:** Natalie García Cam, Alexandra Banda Baltodano, Yaime Condori-Arias, Alvaro Taype-Rondán

**Affiliations:** Department of Otorhinolaryngology. Hospital Nacional Arzobispo Loayza. Lima 15082, Peru; Department of Otorhinolaryngology. Hospital Nacional Arzobispo Loayza. Lima 15082, Peru; Department of Anatomical Pathology. Hospital Nacional Arzobispo Loayza. Lima 15082, Peru; Unidad de Investigación para la Generación y Síntesis de Evidencias en Salud, Vicerrectorado de Investigación, Universidad San Ignacio de Loyola, Lima 15082, Perú; EviSalud - Evidencias en Salud, Lima 15082, Peru

**Keywords:** alveolar rhabdomyosarcoma, paranasal sinuses, parameningeal rhabdomyosarcoma, PAX3-FKHR fusion, case report

## Abstract

Alveolar rhabdomyosarcoma (ARMS) is the most aggressive subtype of rhabdomyosarcoma. Prognosis is closely linked to anatomical location, with parameningeal involvement and distant metastasis being associated with poorer outcomes. Diagnosis is challenging and requires immunohistochemistry, RT-PCR, and FISH. We report the case of a 17-year-old Peruvian male diagnosed with ARMS who presented with multiple adverse prognostic features, including parameningeal–paranasal disease, orbital invasion, distant metastasis, and PAX3–FOXO1 fusion. The clinical trajectory rapidly progressed and the patient succumbed. This case highlights not only the biological aggressiveness of ARMS, but also the systemic delays in diagnosis that may occur in resource-limited settings. Its educational value lies in raising awareness about diagnostic inequity in pediatric oncology and emphasizing the need for early suspicion and timely referral in atypical clinical presentations.

## Introduction

RMS is a malignant neoplasm of primitive mesenchymal origin, and its prognosis is influenced by the tumor location. Parameningeal tumors such as the paranasal sinuses are associated with poor outcomes [1, 2]. RMS represents 5–7% of malignancies in adolescents and young adults, with the alveolar subtype representing the most aggressive histological variant [2, 3].

ARMS has a poorly differentiated cellular morphology that requires confirmatory diagnostic techniques such as immunohistochemistry, RT-PCR, and FISH [4]. These diagnostic requirements can delay diagnosis and treatment [3].

The therapeutic approach to RMS involves multimodal treatment including surgical resection, systemic chemotherapy, and radiotherapy [5]. The Intergroup Rhabdomyosarcoma Study (IRS) has developed risk stratification systems that guide treatment based on tumor location, resectability, histological subtype, and molecular findings [5, 6]. High-risk patients, especially those with metastatic disease, have significantly lower survival rates despite intensive treatment [7]. However, treatment is highly dependent on early diagnosis, which may be delayed in resource-limited and over-burdened settings.

The outcomes of RMS are influenced by prognostic factors including patient age, tumor location, resectability, presence of metastases, histopathological subtype, and tumor biology.

Although the biological aggressiveness of ARMS is well documented, its management in health systems with limited diagnostic infrastructure remains poorly characterized. Few case reports from resource-constrained clinical environments have explored the impact of fragmented access to pathology services and delayed molecular testing on patient outcomes.

In resource-constrained clinical settings, access to advanced diagnostic techniques is often limited, restricting both the accurate classification and timely prognostication of patients with ARMS. In many instances, the diagnostic workup is restricted to immunohistochemistry, whereas more complex analyses must be outsourced to tertiary centers or international laboratories, contributing to delays, as illustrated in this case.

## Case report

**Figure 1:**
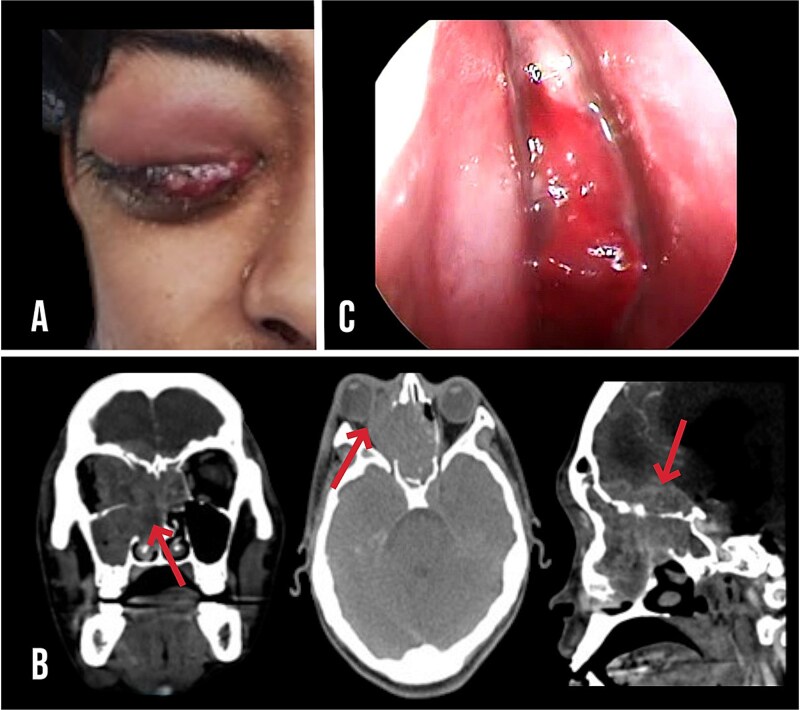
(A) 17-year-old male presenting with proptosis, redness, and swelling of the right eye. (B) CT in coronal, axial and sagittal view, showing a tumour occupying the right frontal and maxillary sinuses, extending into the orbit and causing osteolytic destruction at the anterior skull base with signs of intracranial invasion, respectively (red arrow). (C) Nasoendoscopic image of the tumour at the level of the right inferior turbinate, which was subjected to biopsy.

A 17-year-old Peruvian male presented to the emergency department with a three-week history of right eye irritation that progressed to diplopia, complete vision loss, worsening right temporal headache, vomiting, and significant weight loss. On examination, he exhibited right-sided proptosis, periorbital edema, conjunctival injection, and inability to open the right eye (Fig. 1A). Neurological evaluation revealed no light perception, complete ophthalmoplegia, absence of the right corneal reflex, and hypoesthesia in the ophthalmic branch (V1) of the trigeminal nerve. Bilateral cervical lymphadenopathy was also observed.

Computed tomography (CT) revealed a heterogeneously enhanced mass involving the right frontal and maxillary sinuses with extension into the orbit, osteolytic erosion of the anterior skull base, and signs of intracranial invasion (Fig. 1B).

Nasal endoscopy performed two days after admission revealed a smooth-surfaced mass arising from the inferior turbinate, which was biopsied from the right nasal cavity. Fine-needle aspiration of the cervical lymph nodes was performed (Fig. 1C).

**Figure 2:**
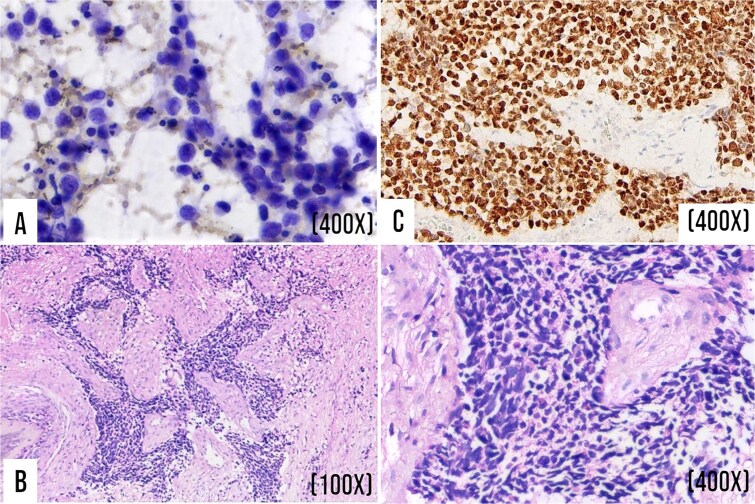
Cytological study of cervical lymphadenopathy, histological study of nasal tissue, and immunohistochemistry to myogenin. (A) Cellular smear obtained by FNA from the right cervical lymph node region demonstrated a neoplastic cell population with round, hyperchromatic nuclei and atypical mitoses. (Papanicolaou stain, 400x). (B) Histological section of nasal biopsy shows a primitive neoplasm composed of round blue cells separated by fibroconnective tissue septa. (Hematoxylin–eosin stain; 100x and 400x). (C) Strong and diffuse positivity for myogenin. (400x).

Histological analysis of the nasal biopsy and lymph node aspirate revealed an infiltrative neoplasm composed of primitive round cells with hyperchromatic nuclei and a scant cytoplasm (Fig. 2A and B). Two weeks after admission, tissue samples were sent to the National Oncology Institute, where immunohistochemistry was performed. The tumor was negative for pancytokeratin, CD45, S100, chromogranin, and NSE. Desmin, myogenin, and MyoD1 showed mild-to-moderate cytoplasmic and nuclear positivity. The mitotic index was approximately 80%, supporting the diagnosis of high-grade malignant neoplasm with rhabdomyoblastic differentiation (Fig. 2C). Post-mortem RT-PCR confirmed the t(2;13)(q35;q14) chromosomal translocation and PAX3–FKHR fusion.

During the second week of hospitalization, the patient developed fever, leukopenia, anemia, and elevated C-reactive protein level. Empirical intravenous ceftriaxone was administered. A bone marrow biopsy confirmed metastatic infiltration. By the third week, the patient was transferred to the National Oncology Institute, where the disease was classified as stage IV, according to the IRS criteria. Given the extent of the orbital, intracranial, nodal, and marrow involvement, surgical resection was not feasible. Palliative treatment with a VAC chemotherapy regimen (vincristine, actinomycin D, and cyclophosphamide) was initiated, and radiotherapy was planned at a total dose of 3900 cGy in 13 sessions. Despite intervention, the patient developed multisystem organ failure and died shortly thereafter.

## Discussion

ARMS is an aggressive subtype of RMS with poor prognosis, particularly in adolescents and in cases involving parameningeal sites, such as the paranasal sinuses. The prognosis worsens with orbital or intracranial extension and unresectable disease [1, 2, 5, 8, 9]. Imaging (MRI/CT) is essential to delineate tumour extent and guide management in such anatomically complex regions [5, 6, 9].

An accurate diagnosis requires a multimodal approach that integrates histopathological, immunophenotypic, and molecular techniques. In the present case, histopathological evaluation revealed a poorly differentiated neoplasm composed of primitive round cells with a scant cytoplasm and hyperchromatic nuclei. This morphology has led to a broad differential diagnosis, including hematolymphoid, neuroectodermal, and undifferentiated epithelial malignancies [8]. Immunohistochemistry played a decisive role: Fig. 2C demonstrates strong nuclear myogenin positivity, confirming rhabdomyoblastic differentiation, while a markedly elevated mitotic index suggested an aggressive biological potential [8, 9].

Although such features may reinforce the clinical urgency, they offer limited prognostic stratification. Molecular confirmation of PAX3–FOXO1 fusion via RT-PCR not only substantiates ARMS diagnosis, but also stratifies prognosis, as this translocation correlates with poorer outcomes relative to PAX7–FOXO1 or fusion-negative variants [5, 9].

In resource-constrained settings, diagnostic work-up is restricted to immunohistochemistry, while immunohistochemical studies were performed locally; however, molecular confirmation required referral to a specialized tertiary center, leading to delays in the final diagnosis. Such barriers may hinder the timely initiation of risk-adapted treatment protocols and potentially compromise patient outcomes [7]. The diagnostic gap described in this case echoes the WHO Essential Diagnostics List framework, which recognizes molecular pathology as critical, yet often unavailable, in underserved regions.

Although aggressive multimodal therapy can improve outcomes in localized ARMS [5], advanced-stage patients may still face a high disease burden, relapse, and death despite treatment [10]. The local failure rate is 16% in patients with bony infiltrative and parameningeal tumours [5].

Beyond the histopathological complexity, this case provides significant educational value. For frontline clinicians, this underscores the need to consider malignancy in children with persistent orbital symptoms, even when initial presentations resemble benign conditions. Early referral and biopsy are critical. For policymakers, this case highlights the urgency of investing in decentralized molecular diagnostics and efficient referral networks to enable timely and equitable oncology care.

## Consent

Written Informed consent was obtained from the patient and his representative for the publication of this case and all accompanying images.

## Guarantor

Dr. Alexandra Banda Baltodano. Department of Otorhinolaryngology. National Hospital Arzobispo Loayza. Lima. 15 082. Peru.
